# Predicting brain metastases of breast cancer based on serum S100B and serum HER2

**DOI:** 10.3892/ol.2013.1536

**Published:** 2013-08-19

**Authors:** TROELS BECHMANN, JONNA SKOV MADSEN, IVAN BRANDSLUND, ERIK DALSGAARD LUND, TINA ORMSTRUP, ERIK HUGGER JAKOBSEN, ANNE MARIE BAK JYLLING, KARINA DAHL STEFFENSEN, ANDERS JAKOBSEN

**Affiliations:** 1 Faculty of Health Sciences, University of Southern Denmark, Odense 5000, Denmark; 2 Department of Oncology, Vejle Hospital, Vejle 7100, Denmark; 3 Department of Biochemistry, Vejle Hospital, Vejle 7100, Denmark; 4 Department of Radiology, Vejle Hospital, Vejle 7100, Denmark; 5 Department of Pathology, Odense University Hospital, Odense 5000, Denmark; 6 Department of Oncology, Odense University Hospital, Odense 5000, Denmark

**Keywords:** breast cancer, brain metastases, serum human epidermal growth factor receptor 2, HER2/neu, serum S100B

## Abstract

Brain metastases are a major cause of morbidity and mortality in breast cancer. The aim of the current study was to evaluate the prediction of brain metastases based on serum S100B and human epidermal growth factor receptor 2 (HER2). A total of 107 breast cancer patients were included in the current study from two prospective cohort studies with either elevated serum HER2 levels >15 ng/ml or brain metastases verified by magnetic resonance imaging (MRI) or computer tomography (CT). Following the exclusion of six patients, the remaining 101 patients were divided into two groups: Group 0 (n=55), patients with normal MRI results; and group 1 (n=46), patients with brain metastases. The levels of serum S100B and HER2 in the two groups were analyzed prior to MRI or CT of the brain, and no significant differences were identified in the serum HER2 (P=0.060) or S100B levels (P=0.623) between the groups. The univariate analysis of prognostic factors for brain metastases showed a significant correlation with systemic disease (P<0.001), axillary lymph node metastases (P=0.001) and serum HER2 >30 ng/ml (P=0.002). Only systemic disease (P<0.001) remained statistically significant in the multivariate analysis. In conclusion, serum levels of S100B and HER2 did not predict the risk of brain metastases. In the multivariate analysis, brain metastases were only found to correlate with systemic disease. However, in the univariate analysis, serum HER2 levels >30 ng/ml were identified to correlate with increased risk of brain metastases, which calls for further investigation.

## Introduction

Breast cancer is the leading malignant disease among females in the industrialized world. Although its incidence has increased over the past decade, there has been a significant decline in mortality from the disease in Denmark, possibly due to advances in screening programs, surgical techniques and adjuvant treatment (1). Despite this progress, a significant number of females experience systemic spread of the disease. Brain metastases, in particular, remain a major cause of morbidity and mortality.

The subset of patients with overexpression of human epidermal growth factor receptor 2 (HER2) has been reported to have a high incidence of brain metastases (25–36%), even following adjuvant treatment with trastuzumab. Risk factors for the development of brain metastases in these patients include, estrogen receptor (ER)-negative tumor tissue, liver metastases, tissue HER2-positive disease and age <50 years (2–4).

At present, breast cancer patients are only evaluated for brain metastases in cases of symptom presentation and the use of magnetic resonance imaging (MRI) for the early diagnosis of subclinical brain metastases remains controversial (5). Miller *et al* found no improvement in overall survival among patients with brain metastases detected by screening (6). However, Niwińska *et al* demonstrated that whole brain radiotherapy reduced the risk of mortality due to progression within the brain from 48 to 16%, comparing symptomatic brain metastases with occult brain metastases detected by MRI screening (7). However, there was no difference in overall survival between the two groups of patients.

Overexpression of the HER2 protein and/or amplification of the HER2 gene is detected in 15–20% of breast cancer tumors, leading to increased tumor cell proliferation, and is associated with aggressive tumor behavior and poor prognosis (8,9). In addition, HER2 overexpression and/or amplification predicts the effect of HER2-targeted therapeutics, including trastuzumab (Herceptin^®^) and lapatinib (Tyverb^®^) in metastatic and adjuvant settings (10–12).

HER2 (neu, ErbB2 or p185HER2) is a tyrosine kinase receptor in the HER family, which includes HER1 (EGFR), HER2, HER3 and HER4. The HER2 gene is located on chromosome 17 and encodes HER2, which is a 185-kDa glycoprotein composed of an intracellular tyrosine kinase domain, a transmembrane domain and an extracellular domain with an unknown ligand (13). Activation of the HER2 pathway is presumably driven by heterodimerization of HER2 with HER1, HER3 or HER4 and the subsequent activation of the downstream pathway (14).

The extracellular domain may be cleaved and measured in serum as ‘serum HER2’ by an enzyme-linked immunosorbent assay (15). The two most common assays used for monitoring serum HER2, HER2/neu ELISA (Oncogene Science, Cambridge, MA, USA) and ADVIA Centaur Serum HER2/neu assay (Siemens Healthcare Diagnostics, Deerfield, IL, USA), have a reference cut-off of 15 ng/ml (16). Carney *et al* demonstrated elevated serum HER2 in 18% (0–38%) of patients with primary breast cancer and in 46% (23–80%) of patients with metastatic breast cancer (17). A number of studies have reported a correlation between elevated levels of serum HER2 and clinical outcome (18–22). In addition, specific studies have reported increasing serum HER2 levels prior to the relapse of breast cancer. However, the potential clinical implications associated with these observations remain to be shown (23–25).

S100B is a calcium binding protein specific to nervous tissue, including glial and Schwann cells. The protein has been revealed as a homo- or heterodimer consisting of two subunits (A and B) and S100B includes S100BB and S100AB. Depending on the concentration, S100B stimulates neurite outgrowth, survival of neurons or the expression of inflammatory cytokines and induces apoptosis (26). Since S100B is a relatively small protein (9–13 kDa), it has been hypothesized to pass through the intact blood-brain barrier. However, elevated S100B is only measured in serum under pathological conditions that also compromise the blood-brain barrier. Serum S100B is measured by immunoassays; however, as there is no established cut-off, it is currently being determined which commercial S100B assays are more accurate (27,28). Yoon *et al* measured serum S100B in 74 healthy controls by the Elecsys S100 Immunoassay. The authors found a reference value for the 95th percentile of 0.12 μg/l, which is in accordance with the cut-off of 0.105 μg/l reported by the manufacturer (29).

The clinical utility of S100B has been evaluated in various studies, indicating a correlation between increased serum S100B and poorer outcome in traumatic brain injury and subarachnoid hemorrhage. A small study of 20 glioma patients by Vos *et al* demonstrated a significantly shorter median survival (25 vs. 38 months) in patients with serum S100B levels >0.09 μg/l (30). A large retrospective multicenter study of 692 malignant melanoma patients found that elevated serum S100B correlated with inferior overall survival, but only in the univariate analysis (31). Serum S100B has also been evaluated as a screening tool for asymptomatic brain metastases in 38 newly diagnosed non-small cell lung cancer patients. The study identified elevated serum S100B (0.28±0.19 μg/l) in all 7 patients with brain metastases identified by MRI (32).

At present, no studies have examined the association between elevated serum S100B and brain metastases or the use of serum S100B as a screening tool for brain metastases in breast cancer. Therefore, it is important to clarify whether elevated serum S100B alone or together with serum HER2 correlates with the incidence of brain metastases to identify patients for later intervention studies testing the clinical effect of early HER2-targeted treatment and radiotherapy. The aim of the present study was to address this issue.

## Patients and methods

### Study population and patient samples

Two cohorts of patients were obtained from two prospective studies (TL and VSL) performed at a single center cancer hospital (Vejle Hospital, Denmark). Serum HER2 levels were analyzed every 6 months during routine follow-up after primary surgery for stage I–IIIA breast cancer or stage IV metastatic breast cancer in a total of 1,308 patients. Patients provided written informed consent. The two studies were approved by the Regional Scientific Ethical Committee for Southern Denmark (project nos. S-VF-20040017 and S-VF-20040101).

**Figure 1:**
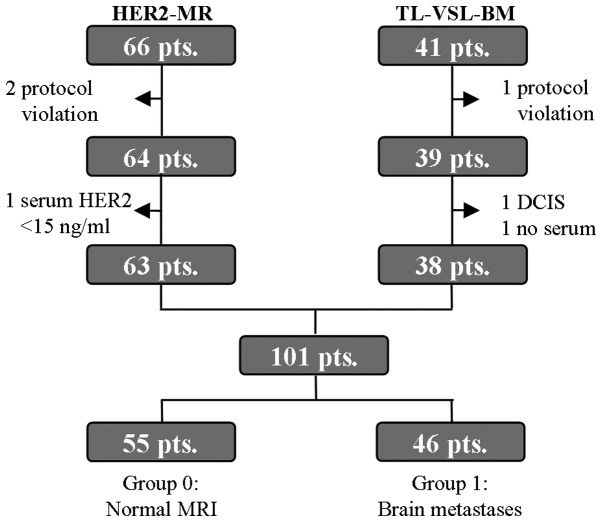
Diagram outlining the exclusion of patients. HER2-MR included 66 patients who underwent brain MRI due to elevated serum HER2 levels >15 ng/ml. TL-VSL-BM included 41 patients treated with radiotherapy for MRI- or CT- verified brain metastases. HER2, human epidermal growth factor receptor 2; MRI, magnetic resonance imaging; CT, computed tomography; DCIS, ductal carcinoma *in situ*.

Sixty-six patients with elevated serum HER2 levels >15 ng/ml during follow-up in the TL and VSL studies were included in the HER2-MR protocol between 15 December, 2010 and 12 April, 2012. Individuals who provided written informed consent and demonstrated no symptoms of brain metastases were eligible. Three patients were excluded due to protocol violation or serum HER2 levels <15 ng/ml at the time of inclusion (Fig. 1). The remaining 63 patients (40 patients in follow-up after primary surgery and 23 with systemic disease) underwent brain MRI and a computed tomography (CT) scan of the thorax and abdomen if in follow-up without relapse. The MRI scans were examined by two dedicated radiologists who reached agreement in all cases. The CT scans were examined in a routine setting. The protocol was approved by the Regional Scientific Ethical Committee for Southern Denmark (project no. S-20100080).

Forty-one patients (referred to as TL-VSL-BM), treated with radiotherapy for MRI- or CT-verified brain metastases between 25 August, 2005 and 20 June, 2011, were included if the individual had received a serum HER2 test under the TL or VSL protocol within 3 months prior to being diagnosed with brain metastases. Three patients were excluded due to protocol violation, only ductal carcinoma *in situ* (DCIS) at the primary surgery or no serum remaining for analysis, leaving 38 patients for further investigation. The additional analysis of serum S100B was also approved by the Regional Scientific Ethical Committee for Southern Denmark.

The remaining 101 patients were divided into two groups: Group 0, the control group (n=55), consisting of patients with normal MRI results and without symptoms of brain metastases; and group 1 (n=46; 38 TL-VSL-BM and 8 HER2-MR patients), comprising patients with MRI- or CT-verified meningeal and/or brain metastases (Fig. 1). The two groups were analyzed for serum HER2 and S100B levels prior to MRI or CT. Serum samples were stored in a local biobank at −80°C.

### Clinical and histopathological data

Histopathological data were obtained from the Danish Breast Cancer Cooperative Group (DBCG) and verified in the local database at the Department of Pathology, Vejle Hospital (Vejle, Denmark). Clinical patient data were obtained from the local electronic health record and complemented with data from the nationwide online electronic health record containing data from all Danish hospitals.

### Biochemical and histopathological methods

Tissue HER2 status was determined on paraffin-embedded tumor tissue by immunohistochemistry (IHC) and fluorescence *in situ* hybridization (FISH). The tumors were considered to be HER2-positive when IHC3+ or IHC2+ with FISH ≥2. IHC analysis was assessed by Herceptest^TM^ (DakoCytomation, Glostrup, Denmark), according to the manufacturer’s instructions. IHC0 and IHC1+ were considered to represent HER2-negative, whereas IHC3+ was defined as HER2-positive. IHC2+ was considered to represent borderline and therefore, to determine HER2 status, the HER2 FISH pharmDx^TM^ kit (DakoCytomation) was used. The threshold for HER2 amplification was a ratio of ≥2.0 between HER2 gene copy number and chromosome 17 centromere.

ER staining was performed on paraffin-embedded tumor tissue using an anti-human ER monoclonal antibody (clone 1D5; DakoCytomation) and visualized by the SuperSensitive^TM^ Polymer-HRP IHC detection system (Biogenex, Fremont, CA, USA). Tumors with nuclei staining ≥10% were considered to represent ER-positive samples according to the contemporary DBCG guidelines.

Serum HER2 was measured using the ADVIA Centaur HER2 Immunoassay (Siemens Healthcare Diagnostics). The assay is an automated sandwich immunoassay using two monoclonal antibodies against the extracellular domain of HER2 to detect serum HER2 by direct chemiluminescent technology (33). The assay was controlled by an in-house serum pool at 8 ng/ml and two commercial controls (Siemens Healthcare Diagnostics) at 14 and 113 ng/ml. The inter-assay coefficients of variation (CV) of these controls were 10.7, 5.8 and 4.6%, respectively.

Serum S100B was measured using the Elecsys S100 Immunoassay (Roche Diagnostics GmbH, Mannheim, Germany). The assay is an automated sandwich immunoassay using two monoclonal antibodies against S100B forming a complex to be measured by direct chemiluminescent technology. Serum specimens were measured according to the manufacturer’s instructions. The lower detection limit was 0.005 μg/l and the assay was controlled by commercial controls at 0.176 and 2.28 μg/l with an inter-assay CV between 1.3 and 3.6%.

### Statistical methods

Statistical analyses were performed using STATA 11 (Statacorp, College Station, TX, USA). Fisher’s exact and Pearson’s χ^2^ tests were used to compare categorical data. Continuous variables were compared using the Mann-Whitney U test. A multivariate logistic regression analysis was used for the prognostic factors of brain metastases with dichotomized exposure variables. P<0.05 was considered to indicate a statistically significant difference.

## Results

### Patient characteristics

Final analysis included 101 patients divided into two groups: Group 0 (control group; n=55), patients with normal MRI results and no symptoms of brain metastases; and group 1 (n=46), patients with MRI- or CT-verified meningeal and/or brain metastases. Table I outlines the patient demographics and clinical characteristics of the two groups. Clinical prognostic factors were significantly better in group 0 when compared with that of group 1 with regard to axillary nodal status (P=0.001) and systemic disease (P<0.001). In addition, an increased number of patients in group 1 compared with that of group 0 had systemic disease at the time of diagnosis. In the two groups, a high proportion of tissue HER2-positive individuals were identified. Similarly, differences in adjuvant and palliative treatment were observed. As expected, a significantly greater number of patients in group 1, when compared with that of group 0, received palliative treatment instead of adjuvant therapy; this was due to a greater number of patients in group 1 exhibiting systemic disease at the time of diagnosis.

### Serum S100B

**Figure 2:**
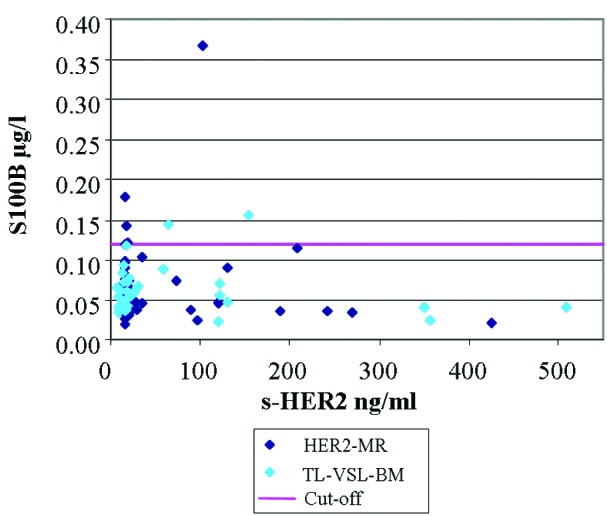
Correlation between serum HER2 and S100B (HER2-MR, n=63; TL-VSL-BM, n=38; cut-off, 0.120 μg/l).

Fig. 2 demonstrates that no correlation was found between serum HER2 and S100B levels with a correlation coefficient (r) of 0.077. Only four out of 63 patients from the HER2-MR protocol had a serum S100B value exceeding the cut-off of 0.120 μg/l. Similarly, only two out of 38 TL-VSL-BM patients had a serum S100B value exceeding the cut-off. Table II demonstrates the sensitivity, specificity and positive and negative predictive value of serum S100B. A total of four out of 46 patients with brain metastases had a serum S100B level exceeding the cut-off of 0.120 μg/l, resulting in a sensitivity of 8.7% (95% CI, 3.2–14.2%).

**Figure 3:**
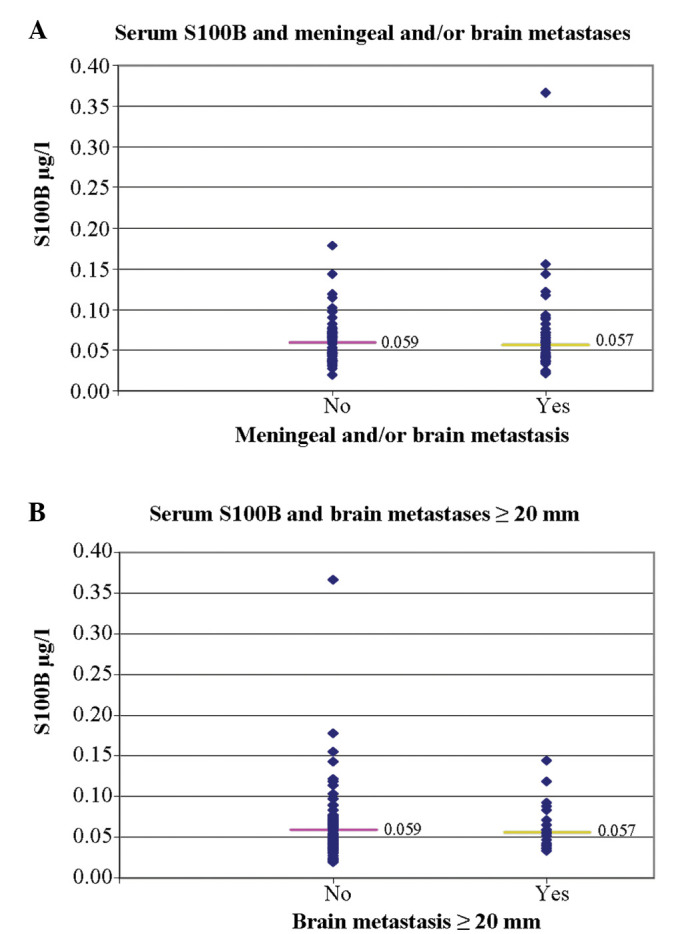
Correlation between (A) serum S100B and meningeal and/or brain metastases and (B) serum S100B and brain metastases ≥20 mm. Medians are presented as purple and yellow.

As presented in Fig. 3A, no significant differences were identified between serum S100B levels of the 46 patients in group 1 with brain metastases (median, 0.057 μg/l; range, 0.021–0.367 μg/l) and the 55 patients in group 0 without brain metastases (median, 0.059 g/l; range, 0.020–0.178 μg/l) (P=0.623). Similarly, no significant differences were identified between the 20 patients with brain metastases >20 mm and the 81 patients with smaller or no brain metastases (P=0.785; Fig. 3B). In addition, no significant differences were identified in serum S100B levels between the 59 patients with systemic disease (median, 0.054 μg/l) and the 42 patients without systemic disease (median, 0.062 μg/l) (P=0.241).

### Serum HER2

No significant differences were identified in serum HER2 levels between the 46 patients in group 1 with brain metastases (median, 21.3 ng/ml; range, 7.6–508.7 ng/ml) and the 55 patients in group 0 without brain metastases (median, 16.5 ng/ml; range, 15.1–207.8 ng/ml) (P=0.0598). This was also true when investigating the difference between the 20 patients with the largest brain metastases, >20 mm, compared with the 81 patients with smaller or no brain metastases (P=0.8579).

The median value of serum HER2 was significantly higher in the 59 patients with systemic disease, 44 with and 15 without brain metastases (median, 21.5 ng/ml), compared with the 42 patients without systemic disease (median, 16.0 ng/ml)(P=0.0002). In addition, serum HER2 was significantly higher in the 31 patients with liver metastases (median, 30.4 ng/ml) than in the 70 patients without liver metastases (median, 16.7 ng/ml) (P=0.0011), and in the 56 tissue HER2-positive patients (median, 19.6 ng/ml) compared with the 45 tissue HER2-negative patients (median, 16.0 ng/ml; P=0.0009) (data not shown).

### Univariate and multivariate analysis of the prognostic factors of brain metastases

In the current study, a univariate analysis of the following variables was performed: Systemic disease (no/yes), age (<60/≥60 years-old), tumor grade (1/2,3 and unknown, with unknown grade corresponding to systemic disease at diagnosis), tumor size (≤20/>20 mm), axillary lymph node metastases (no/yes), ER status (negative/positive), serum HER2 (<30/≥30 ng/ml) and serum S100B (<0.072/≥0.072 μg/l). For serum HER2 and S100B levels, the upper quadrant was compared with lower serum levels, as we hypothesized that the highest serum levels correlated with a poorer outcome and the possible differences, regardless of known cut-off values, were to be analyzed in the present study.

Table III presents the results of the univariate analysis, identifying systemic disease (P<0.001), axillary lymph node metastases (P=0.001) and serum HER2 (P=0.002) as statistically significant prognostic factors of brain metastases. Levels of serum S100B were not statistically significant in the univariate analysis (P=0.662).

The multivariate analysis was performed with the four variables from the univariate analysis that resulted in P<0.100. Only systemic disease (P<0.001) remained an independent prognostic factor of brain metastases, whereas tumor grade (P=0.095), axillary lymph node metastases (P=0.113) and serum HER2 (P=0.894) were not statistically significant in the multivariate analysis.

## Discussion

In the current study, an extremely low number of patients had serum S100B levels exceeding the cut-off value. Subsequently, there was no difference in serum S100B between the patients with and without brain metastases. In addition, patients with the largest brain metastases, >20 mm and smaller or no brain metastases were not found to have different S100B serum levels. The comparison was based on the assumption that the largest brain metastases would cause the greatest damage to the brain tissue and the blood-brain barrier and subsequently have the highest levels of serum S100B. These observations may have several explanations.

Eigentler *et al* reported that serum S100B may be elevated in patients with brain metastases from malignant melanoma (31). However, in contrast to breast cancer, there is an overexpression of S100B in melanoma cells, which may explain the higher level of serum S100B in the brain metastases from malignant melanoma (34). In addition, Vos *et al* reported elevated serum S100B levels in patients with poorer outcome of primary gliomas of the brain as S100B is also expressed in gliomas (30). Korfias *et al* demonstrated elevated serum S100B in patients with traumatic head injury, where more diffuse damage to the glial cells is expected when compared with that of the damage caused by relatively slow growing metastases from breast cancer (26).

The current study did not find a significant difference in the levels of serum HER2 between patients with and without brain metastases; however, in the univariate analysis, serum HER2 levels >30 ng/ml were identified as a prognostic factor of brain metastases. These observations are consistent with a study by Sørensen *et al* reporting that the predictive value of serum HER2 to systemic relapse may be optimized with a cut-off value between 25 and 32 ng/ml in tissue HER2-negative and -positive patients, respectively (25). This may explain why no relapses in the group of patients were reported during the follow-up, as serum HER2 levels were only slightly >15 ng/ml.

The lack of differences in serum HER2 levels between patients with and without brain metastases may be the result of performing only one MRI screen during the current study in connection with elevated serum HER2 levels in the HER2-MR protocol. Therefore, it is possible that the new, smallest brain metastases, not yet visible by MRI, were not detected, but elevated the serum HER2 levels. However, we would expect relapse in certain cases if there was a strong correlation, as the mean time from measurement of elevated serum HER2 to MRI in this study was 71±58 days (standard deviation). In future studies, sequential MRI must be performed to identify a possible lead time from elevated serum HER2 levels to visible metastases by MRI, provided that the smallest brain metastases have the capacity to cause elevated serum HER2.

In the HER2-MR protocol, meningeal and/or brain metastases were identified in eight out of 23 patients with known systemic disease and six of these had tissue HER2-positive disease. In future studies, when evaluating MRI screening for brain metastases, it may be advantageous to focus on the tissue HER2-positive patients with the highest serum HER2 levels in cases of otherwise stable systemic disease. Alternatively, all patients with tissue HER2-positive systemic disease must be offered MRI screening for brain metastases, as it is anticipated that, in the near future, improved targeted therapies are likely to be offered to these patients.

In conclusion, the present study demonstrates that quantitative measurement of serum S100B and serum HER2 cannot be used to identify patients with an increased risk of brain metastases. However, in the univariate analysis, serum HER2 levels >30 ng/ml were found to correlate with an increased risk of brain metastases, which warrants further investigation.

## Figures and Tables

**Table I:** Patient demographics and clinical characteristics.

	Group 0^a^ (n=55)		Group 1^b^ (n=46)		
			
Characteristic	n	%	n	%	P-value
Age, years					
<40	3	5.5	6	13.0	
40–59	35	63.6	23	50.0	
≥60	17	30.9	17	37.0	0.238
Type of surgery					
Breast conserving	37	67.3	14	30.4	
Mastectomy	12	21.8	16	34.8	
Neoadjuvant chemo	3	5.5	1	2.2	
Primary systemic BC	3	5.5	15	32.6	**<0.001**
Tumor type					
Ductal	46	83.6	33	71.7	
Lobular	1	1.8	2	4.3	
Others^c^	8	14.5	11	23.9	0.372
Tumor grade					
1	9	16.4	2	4.3	
2	20	36.4	17	37.0	
3	18	32.7	15	32.6	
Unknown^c^	8	14.5	12	26.1	0.170
Tumor size					
T1	22	40.0	16	34.8	
T2	31	56.4	24	52.2	
T3	2	3.6	6	13.0	0.254
Nodal status					
N0	27	49.1	8	17.4	
N1	15	27.3	9	19.6	
N2	4	7.3	11	23.9	
N3	4	7.3	7	15.2	
Multiple on US/CT^d^	5	9.1	11	23.9	**0.001**
ER status					
Negative	18	32.7	17	37.0	
Positive	37	67.3	29	63.0	0.407
HER2 IHC/FISH					
Negative	25	45.5	20	43.5	
Positive	30	54.5	26	56.5	1.000
Systemic disease					
No	40	72.7	2	4.3	
Yes	15	27.3	44	95.7	**<0.001**

Groups were compared by Fisher’s exact test. Bold P-value denotes statistical signficance.

a Normal magnetic resonance imaging results (control group);

b meningeal and/or brain metastases;

c mainly needle biopsy patients with primary systemic BC and no determination of tumor subtype;

d diagnosis of multiple pathological axillary lymph nodes by US or CT.

Tumor size: T1, ≤20 mm; T2, >20 but ≤50 mm; T3, >50 mm. Nodal status: N0, 0 nodes; N1, 1–3 nodes; N2, 4–9 nodes; N3, ≥10 nodes. BC, breast cancer; US, ultrasound; CT, computed tomography; ER, estrogen receptor; HER2, human epidermal growth factor receptor 2; IHC, immunohistochemistry; FISH, fluroescence *in situ* hybridization.

**Table II:** Serum S100B prior to CT or MRI of the brain.

Serum S100B	Group 0^a^	Group 1^b^	Total
Elevated (>0.120 μg/l)	2	4	6
Normal (≤0.120 μg/l)	53	42	95
Total	55	46	101

a Normal MRI results (control group);

b meningeal and/or brain metastases.

CT, computed tomography; MRI, magnetic resonance imaging.

**Table III:** Univariate analysis of prognostic factors of brain metastasis.

Factor	P-value
Systemic disease, no/yes	<0.001
Age, </≥60 years	0.522
Tumor grade, </≥grade 2^a^	0.054
Tumor size, ≤/>20 mm	0.590
Lymph nodes, −/+	0.001
ER status, −/+	0.656
HER2 IHC/FISH, −/+	0.842
Serum HER2, </≥30 ng/ml	0.002
S100B, </≥0.072 μg/l	0.662

a Grade 2, 3 and unknown.

ER, estrogen receptor; HER2, human epidermal growth factor receptor 2; IHC, immunohistochemistry; FISH, fluroescence *in situ* hybridization.
